# Proprioceptive neuromuscular facilitation improves tendon-bone healing in a rat model of rotator cuff tear: a comparative study with traditional electroacupuncture and infrared therapy

**DOI:** 10.3389/fphys.2026.1879897

**Published:** 2026-07-06

**Authors:** Zhiru Liang, Weihong Chen, Jingyun Kang, Bo Tang, Lei Huang, Peng Liu

**Affiliations:** 1 Department of Hepatobiliary Surgery, Xilingol League Central Hospital, Xilin Hot, Inner Mongolia, China; 2 Department of Central Laboratory, Anxi County Hospital, Quanzhou, Fujian, China; 3 Departmentof Rehabilitation Medicine, Xilingol League Central Hospital, Xilin Hot, Inner Mongolia, China; 4 Department of Neurosurgery, Xilingol League Central Hospital, Xilin Hot, Inner Mongolia, China

**Keywords:** collagen remodeling, electroacupuncture, proprioceptive neuromuscular facilitation (PNF), rehabilitation, rotator cuff tear, tendon-bone healing

## Abstract

**Background:**

To compare the therapeutic effects of proprioceptive neuromuscular facilitation (PNF) versus traditional rehabilitation (electroacupuncture combined with infrared irradiation) on tendon-bone healing in a rat model of rotator cuff tear.

**Methods:**

Thirty-two male SD rats were randomly divided into four groups (n=8 each): sham operation (Sham), rotator cuff tear model without treatment (Model), traditional treatment (Traditional: electroacupuncture + infrared), and PNF treatment (PNF: daily passive stretching with resistance). The rotator cuff tear model was established by transverse incision of 1/2 of the supraspinatus tendon. After 4 weeks of intervention, tendon-bone healing was evaluated by biomechanical testing (maximum failure load), ELISA (IL-1β, IL-6, TNF-α), qPCR (inflammatory gene expression), histology (H&E, Safranin O, Sirius Red, Masson’s trichrome), immunohistochemistry (collagen type I), and Western blot (VEGF, TGF-β).

**Results:**

PNF treatment significantly increased the maximum failure load of the tendon-bone interface compared to the Model and Traditional groups (p < 0.05). Both PNF and Traditional treatments reduced pro-inflammatory cytokine levels (IL-1β, IL-6, TNF-α) at both protein and mRNA levels, with PNF showing a more pronounced anti-inflammatory effect. Histologically, PNF group exhibited better-organized collagen fibers (Masson’s trichrome), increased fibrocartilage regeneration (Safranin O), and a higher ratio of mature type I to type III collagen (Sirius Red under polarized light) compared to the Traditional group. Immunohistochemistry revealed stronger type I collagen deposition in the PNF group. Western blot demonstrated that PNF upregulated VEGF and TGF-β expression more effectively than Traditional treatment.

**Conclusion:**

PNF therapy outperforms traditional electroacupuncture combined with infrared therapy in promoting tendon-bone healing after rotator cuff tear in rats, as evidenced by superior biomechanical strength, enhanced collagen maturation, and better modulation of inflammation and growth factors. PNF is a promising non−invasive rehabilitation approach that warrants further clinical investigation in patients with chronic rotator cuff injury.

## Introduction

Rotator cuff tear (RCT) is one of the most common musculoskeletal disorders affecting the shoulder joint, accounting for a substantial proportion of all shoulder pathologies and representing a major cause of shoulder pain and functional disability worldwide (Fang et al., 2026; Li et al., 2026). The supraspinatus tendon is the most frequently involved site, and the incidence of RCT increases progressively with age, posing a growing public health burden as the population ages (Baek et al., 2026; Kim and Yoon, 2026). Although surgical repair is indicated for massive full-thickness tears, the majority of patients with partial-thickness or chronic tears are managed conservatively. However, current non-surgical treatments—including physical modalities, acupuncture, and exercise therapy—yield inconsistent outcomes, and the optimal rehabilitation strategy for chronic RCT remains poorly defined (Trygonis et al., 2026). Therefore, elucidating the comparative efficacy of different rehabilitation approaches and their underlying mechanisms is of great clinical importance.

Proprioceptive neuromuscular facilitation (PNF) is a therapeutic technique originally developed for neurological rehabilitation, which stimulates proprioceptors to enhance neuromuscular control, muscle strength, coordination, and joint range of motion (Kirkaya et al., 2025). PNF employs spiral and diagonal movement patterns with rhythmic stabilization and resistance, promoting reciprocal inhibition and relaxation of agonist-antagonist muscle pairs (Tang et al., 2026). In recent years, PNF has been increasingly applied to musculoskeletal disorders, particularly shoulder instability and impingement syndromes, where it improves scapular positioning, reduces pain, and restores motor function (Zhu et al., 2025). Electroacupuncture combined with infrared irradiation (EA+IR) is a commonly used non−surgical rehabilitation protocol for chronic rotator cuff injury in traditional Chinese medicine−based practice, serving as an appropriate representative of traditional passive modalities (Wang et al., 2025). For rotator cuff injuries, PNF training targeting shoulder flexion-abduction-external rotation and extension-adduction-internal rotation patterns activates the supraspinatus, infraspinatus, subscapularis, and deltoid muscles, potentially enhancing tendon-bone healing through improved mechanical loading and neuromuscular re-education (Yin et al., 2025).

Traditional Chinese rehabilitation for RCT typically combines electroacupuncture (EA) and infrared irradiation(IR) (Hu et al., 2024). Acupuncture at acupoints such as Jianqian (Extra), Jianzhong (Extra), and Jianzhen (SI9) is believed to promote local blood circulation, reduce inflammation, and alleviate pain (Wang and Zhang, 2026). EA with dense-sparse waves (2/100 Hz) has been shown to modulate inflammatory cytokine expression and promote tissue repair in animal models of tendon injury (Song et al., 2020). Infrared therapy provides deep heating, increases local blood flow, and facilitates tissue relaxation (Singh et al., 2026). Despite their widespread clinical use, the comparative effectiveness of PNF versus traditional EA plus infrared therapy on tendon-bone healing, biomechanical properties, and inflammatory regulation has not been systematically evaluated in a controlled animal study.

Both PNF and traditional rehabilitation are thought to exert therapeutic effects through modulation of local inflammation and promotion of collagen remodeling, but the extent to which these two approaches differ in their impact on key healing parameters remains unknown. To date, no study has directly compared an active mechanotherapy (PNF) with a passive physical modality (EA+IR) on tendon-bone healing outcomes, and this comparison addresses a clinically relevant gap in non-operative management of rotator cuff tears. Connective tissue growth factor, transforming growth factor-beta (TGF-β), and vascular endothelial growth factor (VEGF) are critical regulators of tendon-bone healing, influencing collagen synthesis, angiogenesis, and matrix remodeling (Guo et al., 2024). Pro-inflammatory cytokines such as interleukin-1β (IL-1β), interleukin-6 (IL-6), and tumor necrosis factor-alpha (TNF-α) are elevated in the joint fluid following rotator cuff injury and correlate with pain and healing outcomes. Whether PNF and traditional rehabilitation differentially regulate these molecular mediators has not been directly compared (Guan et al., 2020).

Therefore, the present study was designed to compare the therapeutic effects of PNF versus traditional EA combined with IR on tendon-bone healing in a rat model of rotator cuff tear. We evaluated biomechanical properties, histological outcomes, inflammatory cytokine expression, growth factor expression, and collagen type I deposition after four weeks of intervention. By systematically comparing these two rehabilitation approaches, we aimed to provide experimental evidence for the selection of optimal non-surgical treatment for chronic rotator cuff injury.

## Methods

### Animals and experimental design

All animal procedures were approved by the Ethics Committee of Xilingol League Central Hospital (Approval No. XMZXYYLLWYH2025-046) and were conducted in accordance with the National Institutes of Health Guide for the Care and Use of Laboratory Animals. Thirty-two male Sprague-Dawley rats (6 weeks old, weighing 180–220 g) were used in this study. The animals were housed under specific pathogen-free conditions with a 12-h light/dark cycle at 22–25°C and 50%–60% relative humidity, and had free access to standard chow and sterilized water. After 5 days of acclimatization, the rats were randomly divided into four groups (n = 8 each) using a random number table: sham operation (Sham), rotator cuff tear model without treatment (Model), traditional treatment (Traditional: EA + IR), and PNF treatment (PNF).

### Establishment of rotator cuff tear model

Rats were fasted for 12 h with free access to water before surgery. Anesthesia was induced by intraperitoneal injection of 3% sodium pentobarbital (30 mg/kg). After loss of the righting reflex and pain response, the right shoulder region was shaved and disinfected three times with iodine and 75% ethanol. A 1.5 cm longitudinal skin incision was made over the acromion, and the superficial fascia and trapezius muscle were bluntly dissected to expose the supraspinatus tendon and its insertion on the greater tubercle of the humerus. Under an operating microscope, approximately half of the supraspinatus tendon was transversely cut using micro-scissors. The wound was irrigated with sterile saline and hemostasis was achieved by gentle compression. After confirming the absence of active bleeding, the muscle, fascia, and skin were sutured layer by layer with 5–0 silk, and the incision was disinfected again with iodine. For the sham group, the skin, fascia, and trapezius muscle were incised to expose the supraspinatus tendon without cutting it, followed by the same closure and disinfection procedures. Postoperatively, rats were housed individually for 3 days to prevent wound biting, and penicillin sodium (200,000 U/kg) was injected intramuscularly daily for 3 days to prevent infection. Successful model establishment was confirmed by reduced food intake, limping of the right forelimb, and reluctance to bear weight, which persisted after recovery of food intake.

### Intervention protocols

All treatments were initiated on the day after successful modeling, performed once daily, 6 days per week with 1 day of rest, for a total of 4 weeks.

PNF group: The rat was gently fixed by the trunk while the right shoulder joint was stabilized. Passive stretching was sequentially applied in six directions: flexion, abduction, extension, adduction, internal rotation, and external rotation. In each direction, moderate resistance was applied, and the stretch was maintained for 5–8 s followed by 5 s of relaxation. Each movement was repeated for 10 cycles, with a total session duration of 30 min. To ensure consistency, the resistance was applied by a single trained operator at an intensity just sufficient to prevent passive movement without causing visible distress, and the same sequence of six directions was followed for each rat daily. Unlike simple passive stretching, the PNF protocol applied a diagonal−spiral pattern with moderate manual resistance during the end range of each motion, with the intensity set just sufficient to prevent further passive movement without causing distress, consistent with the rhythmic stabilization principle of PNF.

Traditional group: IR was applied to the right shoulder from a distance of approximately 20 cm for 20 min, with non-target areas shielded to prevent burns. Immediately after irradiation, EA was administered at three acupoints (Jianqian, Jianzhong, Jianzhen) on the right shoulder. After routine disinfection, disposable acupuncture needles (0.20 mm × 15 mm) were inserted to a depth of 0.5 cm. The needles were connected to an EA device (G6805-2A) using a dense-sparse wave (2/100 Hz) with an intensity of 0.5–1 mA, adjusted to induce mild rhythmic muscle twitching. Each EA session lasted 20 min.

Model and Sham groups: Rats were housed under the same conditions without any rehabilitation or physical intervention.

### Specimen collection and preparation

At week 4 after modeling, rats were euthanized by an overdose of 3% sodium pentobarbital (50 mg/kg). The right shoulder joint was re-opened, and the supraspinatus muscle–humerus complex was carefully exposed. The joint capsule and synovium were excised, placed in ice-cold PBS (pH 7.4), and centrifuged at 3000 r/min for 15 min at 4°C. The supernatant was collected and stored at –80°C for ELISA. The supraspinatus muscle–humerus complex was then isolated by removing all other muscles and connective tissues.

### Biomechanical testing

Samples stored at –40°C were thawed at room temperature for 30 min, rinsed with saline, and carefully cleaned of residual soft tissue while preserving the tendon–bone interface. Each specimen was mounted on an ElectroForce 3200 biomechanical testing machine using custom fixtures, with the humerus fixed by a metal clamp and the tendon secured by a mesh anti-slip clamp. The pulling direction was aligned with the tendon fibers. After preloading with 2 N for 20 s repeated three times to eliminate viscoelasticity, a tensile test was performed at a speed of 5 mm/min with a sampling frequency of 100 Hz until complete failure of the tendon–bone interface. The maximum failure load (N) was recorded from the load–displacement curve. Biomechanical testing was performed by an investigator blinded to the group allocation.

### Enzyme-linked immunosorbent assay

The concentrations of IL-1β, IL-6, and TNF-α in joint fluid supernatants were measured using rat-specific ELISA kits (Shanghai Jianglai Biotechnology) according to the manufacturer’s instructions. Briefly, 50 μL of standard or sample was added to pre-coated wells, followed by 50 μL of HRP-conjugated detection antibody. After incubation at 37°C for 30 min, the plates were washed five times with washing buffer. Substrates A and B (50 μL each) were added and incubated in the dark at 37°C for 10 min. The reaction was stopped by adding 50 μL of stop solution, and the optical density was read at 450 nm using a microplate reader. Standard curves were constructed using four-parameter logistic regression, and cytokine concentrations were expressed as pg/mL.

### Histological staining

Fixed specimens were decalcified in 10% EDTA (pH 7.4) for 4–6 weeks at room temperature, with the decalcifying solution changed every 5 days until a needle could be easily inserted into the cancellous bone. After decalcification, tissues were dehydrated through graded ethanol (70%, 80%, 95%, 100%), cleared in xylene, and embedded in paraffin. Serial sections (5–7 μm) were cut along the sagittal plane of the supraspinatus tendon–humerus complex.

Hematoxylin and eosin (H&E) staining was performed to evaluate inflammation, cell structure, and tissue integrity. Sections were deparaffinized, rehydrated, stained with hematoxylin for 5 min, differentiated in 1% acid alcohol for 2 s, blued in running water for 10 min, and counterstained with eosin for 2 min. Masson’s trichrome staining was used to assess collagen distribution and fibrosis. Sections were stained with hematoxylin, followed by Ponceau-acid fuchsin, phosphomolybdic acid, and aniline blue. Safranin O-fast green staining was performed to evaluate fibrocartilage regeneration. Sections were stained with hematoxylin, fast green for 5 min, and safranin O for 5 min. Sirius red staining was used to analyze collagen type and maturity. Sections were stained with 0.1% Sirius red in saturated picric acid for 30 min, then washed with 0.5% acetic acid. Sirius red-stained sections were examined under polarized light to distinguish type I (yellow/red) and type III (green) collagen. Histological scoring for H&E, Safranin O, and Sirius red was performed using a semi-quantitative scale (0–12 or 0–10) based on cell arrangement, fiber continuity, interface integrity, and inflammatory cell infiltration. Quantitative analysis of Masson-stained sections was performed by calculating the collagen area fraction using ImageJ software.

### Immunohistochemistry

After deparaffinization and rehydration, sections were treated with 3% H_2_O_2_ for 10 min to block endogenous peroxidase. Antigen retrieval was performed using citrate buffer (pH 6.0) under high pressure for 3 min. After blocking with goat serum for 30 min, sections were incubated with rabbit anti-rat Col I antibody (CST, 1:200) overnight at 4°C. After washing with PBS, sections were incubated with HRP-conjugated secondary antibody for 30 min at 37°C. Immunoreactivity was visualized using DAB substrate, and sections were counterstained with hematoxylin. The positive area and integrated optical density (IOD) were quantified using ImageJ software.

### Western blot

Total protein was extracted from frozen supraspinatus muscle tissue using RIPA lysis buffer containing PMSF. Protein concentration was determined using the BCA method. Equal amounts of protein (30 μg) were separated by 10% SDS-PAGE and transferred onto PVDF membranes. After blocking with 5% non-fat milk for 1 h at room temperature, membranes were incubated overnight at 4°C with primary antibodies against VEGF (1:1000), TGF-β (1:1000), and GAPDH (1:5000). After washing with TBST, membranes were incubated with HRP-conjugated secondary antibody (1:5000) for 1 h at room temperature. Protein bands were visualized using ECL chemiluminescence reagent and captured with a gel imaging system. Band intensities were quantified using ImageJ software and normalized to GAPDH.

### Quantitative real-time PCR

Total RNA was extracted from frozen tissue using TRIzol reagent. RNA purity and concentration were assessed by spectrophotometry (A260/A280 ratio between 1.8 and 2.0). Reverse transcription was performed using PrimeScript RT Master Mix (TaKaRa) following the manufacturer’s protocol. qPCR was carried out using TB Green Premix Ex Taq II (TaKaRa) on a 7500 Real-Time PCR System (Applied Biosystems). The primer sequences were as follows: IL-1β forward 5′-GAAATGCTGCTGAGGAAGATGC-3′, reverse 5′-GTCGTTGCTTCTGTCTTCCTG-3′; IL-6 forward 5′-CCGGAGAGGAGACTTCACAG-3′, reverse 5′-CAGAATTGCCATTGCACAACTC-3′; TNF-α forward 5′-CCTCTCTCTAATCAGCCCTCTG-3′, reverse 5′-GAGGACCTGGGAGTAGATGAG-3′; and GAPDH forward 5′-TGCCCGTCTCTGGTGGTG-3′, reverse 5′-CCTTGCTCAGTGTCCTTGCTG-3′. The cycling conditions were: 95°C for 30 s, followed by 40 cycles of 95°C for 5 s and 60°C for 30 s. Relative gene expression was calculated using the 2^^(-ΔΔCt)^ method with GAPDH as the internal reference.

### Statistical analysis

All data were expressed as mean ± standard deviation (SD). Statistical analyses were performed using SPSS 25.0 software. Comparisons among multiple groups were conducted using one-way analysis of variance (ANOVA) followed by Tukey’s *post-hoc* test. Biomechanical testing, qPCR, and ELISA used all eight rats per group (n=8). For histology and Western blot, three rats per group were randomly selected as a sub-sample, as *a priori* power analysis indicated that n=3 was sufficient to detect the expected differences. A value of *P* < 0.05 was considered statistically significant.

## Results

### Establishment of rotator cuff tear model

**Figure 1:**
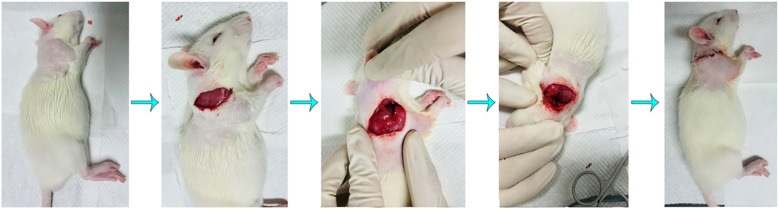
Schematic diagram of the rotator cuff tear model establishment. The surgical procedure included skin incision, exposure of the supraspinatus tendon, transverse incision of approximately half of the tendon, hemostasis, and layered suture.

The surgical procedure for establishing the rotator cuff tear model is illustrated in **Figure 1**. After anesthesia and disinfection, a 1.5 cm longitudinal incision was made over the right acromion, and the supraspinatus tendon was exposed. Approximately half of the supraspinatus tendon was transversely cut, followed by hemostasis and layered suture. Postoperatively, rats in the model group exhibited reduced food intake, limping of the right forelimb, and reluctance to bear weight, confirming successful modeling. Sham-operated rats underwent the same procedure without tendon cutting.

### PNF treatment improved biomechanical strength and collagen organization

**Figure 2:**
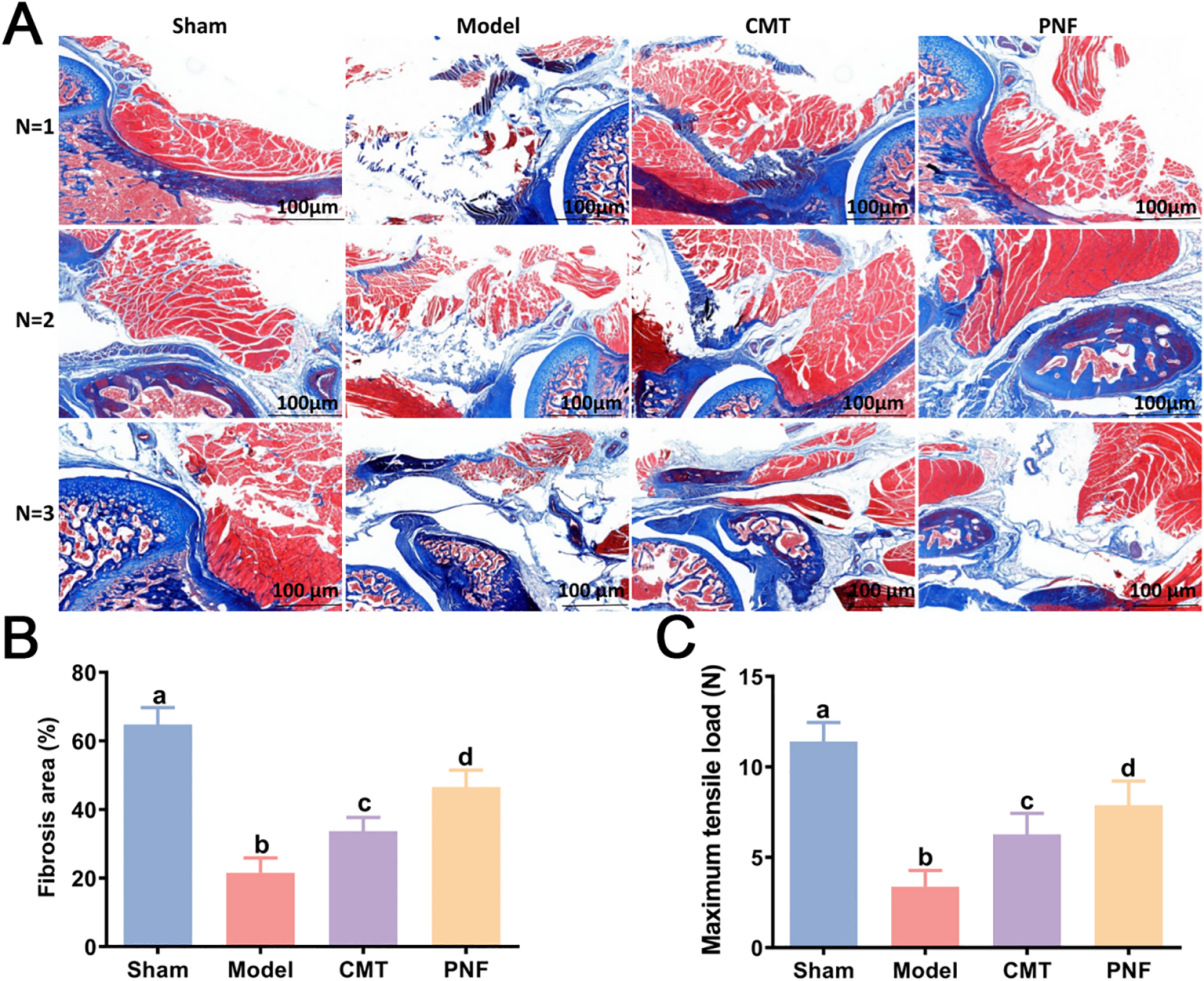
Effects of PNF and traditional treatments on collagen organization and biomechanical properties. **(A)** Representative images of Masson’s trichrome staining of the tendon-bone interface in the Sham, Model, Traditional, and PNF groups. Scale bar = 100 μm. **(B)** Quantitative analysis of collagen area fraction (%). **(C)** Maximum failure load (N) measured by biomechanical testing. Data are presented as mean ± SEM (n = 3 per group for histology; n = 8 per group for biomechanics). Statistical analysis was performed using one-way ANOVA followed by Tukey’s *post-hoc* test. Different letters (a, b, c, d) indicate statistically significant differences at *P* < 0.05 between groups.

The effects of different rehabilitation interventions on tendon-bone healing were evaluated by Masson’s trichrome staining and biomechanical testing (**Figure 2**). Masson’s trichrome staining (**Figure 2A**) showed that the Model group exhibited disorganized collagen fibers with sparse blue staining, indicating poor collagen deposition. The Traditional group showed moderate improvement with more organized collagen bundles. The PNF group displayed densely packed, well-aligned blue-stained collagen fibers, resembling the Sham group. Quantitative analysis of the collagen area fraction (**Figure 2B**) revealed that the PNF group had significantly higher collagen content compared to the Model and Traditional groups. Biomechanical testing (**Figure 2C**) demonstrated that the maximum failure load of the tendon-bone interface was significantly reduced in the Model group compared to the Sham group. Both Traditional and PNF treatments significantly increased the failure load, with the PNF group showing the greatest improvement.

### PNF treatment suppressed inflammatory cytokine expression at protein and mRNA levels

**Figure 3:**
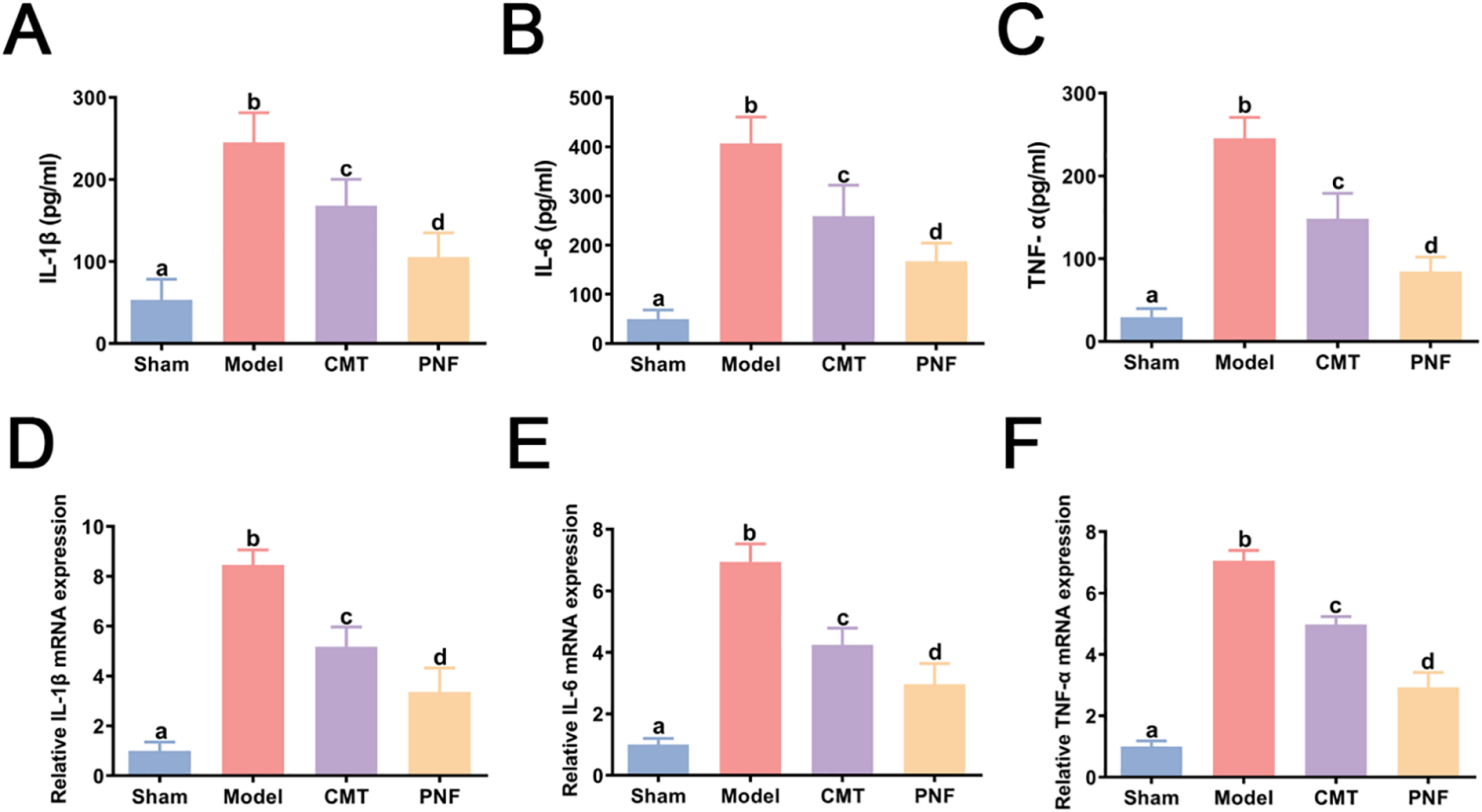
Effects of PNF and traditional treatments on inflammatory cytokine expression. **(A–C)** ELISA quantification of IL-1β, IL-6, and TNF-α protein levels in joint fluid. **(D–F)** qPCR analysis of IL-1β, IL-6, and TNF-α mRNA expression in supraspinatus muscle tissue. Data are presented as mean ± SEM (n = 8 per group for ELISA and qPCR). Statistical analysis was performed using one-way ANOVA followed by Tukey’s *post-hoc* test. Different letters (a, b, c, d) indicate statistically significant differences at *P* < 0.05 between groups.

The protein levels of pro-inflammatory cytokines in joint fluid were measured by ELISA (**Figures 3A–C**). The Model group exhibited significantly elevated IL-1β, IL-6, and TNF-α levels compared to the Sham group. Both Traditional and PNF treatments significantly reduced these cytokines, with the PNF group showing lower levels than the Traditional group. Consistent with the ELISA data, qPCR analysis of supraspinatus muscle tissue (**Figures 3D–F**) revealed that the mRNA expression of IL-1β, IL-6, and TNF-α was markedly upregulated in the Model group. Traditional and PNF treatments both significantly downregulated these inflammatory gene transcripts, and the PNF group exhibited the lowest expression levels.

### PNF treatment improved histological scores and collagen maturation

**Figure 4:**
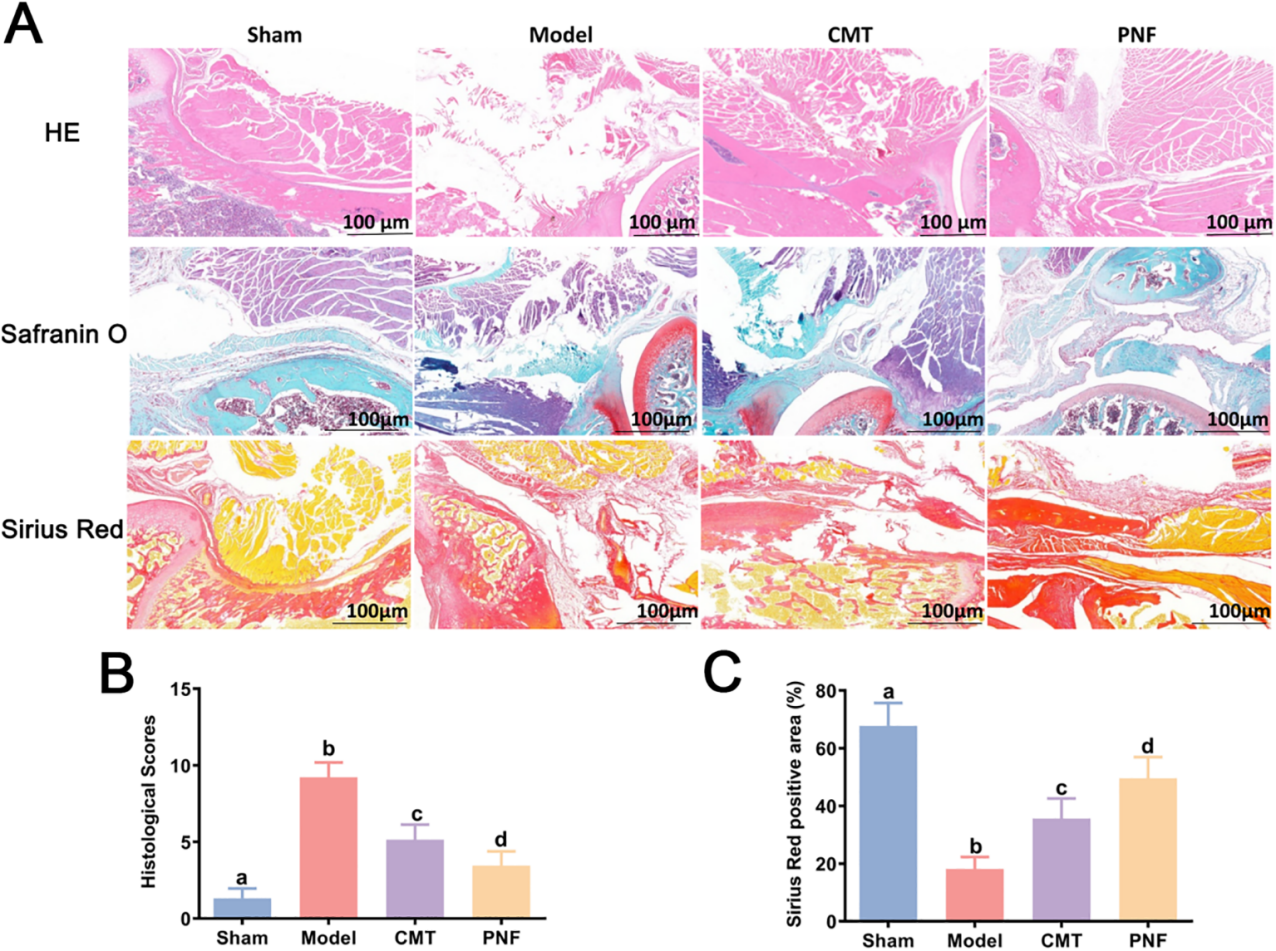
Histological assessment of tendon-bone healing. **(A)** Representative images of H&E staining (top row), Safranin O-fast green staining (middle row), and Sirius red staining under polarized light (bottom row) of the tendon-bone interface. Scale bar = 100 μm. **(B)** Quantitative histological scores for H&E staining (scale 0–12). **(C)** Quantitative analysis of the type I/type III collagen ratio based on Sirius red staining under polarized light. Data are presented as mean ± SEM (n = 3 per group). Different letters (a, b, c, d) indicate statistically significant differences at *P* < 0.05 between groups (one-way ANOVA with Tukey’s *post-hoc* test).

Histological assessment was performed using H&E, Safranin O, and Sirius red staining (**Figure 4A**). H&E staining showed that the Model group had severe inflammatory cell infiltration, disrupted tissue architecture, and poor tendon-bone interface continuity. The Traditional group showed moderate improvement, while the PNF group exhibited well-organized tissue with minimal inflammation, resembling the Sham group. Safranin O staining revealed that fibrocartilage regeneration at the tendon-bone interface was markedly reduced in the Model group, partially restored in the Traditional group, and nearly normalized in the PNF group. Sirius red staining under polarized light showed that the Model group predominantly contained green-stained (type III) collagen, indicating immature scar tissue. The Traditional group showed a mixture of green and yellow/red fibers, whereas the PNF group exhibited abundant yellow/red (type I) collagen, indicating mature and well-organized matrix. Quantitative histological scores (**Figure 4B**) confirmed that the PNF group had significantly higher scores than the Model and Traditional groups. Quantitative analysis of Sirius red-stained sections (**Figure 4C**) demonstrated that the PNF group had a significantly higher type I/type III collagen ratio compared to the Traditional and Model groups.

### PNF treatment enhanced type I collagen deposition

**Figure 5:**
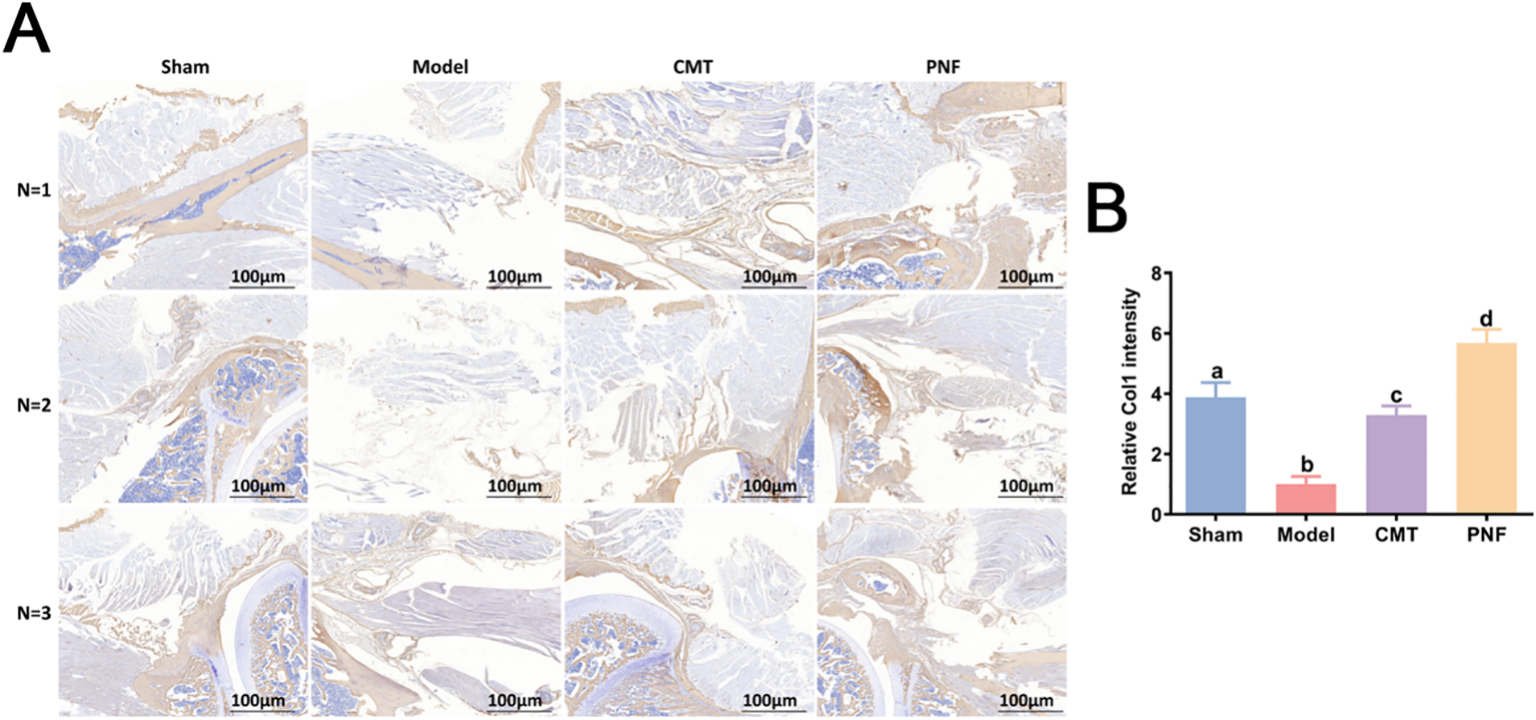
Immunohistochemical detection of type I collagen (Col I) at the tendon-bone interface. **(A)** Representative images of Col I immunohistochemical staining (brown) in each group. Scale bar = 100 μm. **(B)** Quantitative analysis of integrated optical density (IOD) of Col I staining. Data are presented as mean ± SEM (n = 3 per group). Different letters (a, b, c, d) indicate statistically significant differences at *P* < 0.05 between groups (one-way ANOVA with Tukey’s *post-hoc* test).

Immunohistochemical staining for type I collagen (Col I) was performed to assess collagen maturation at the tendon-bone interface (**Figure 5A**). The Model group showed weak and scattered Col I immunoreactivity. The Traditional group exhibited moderate positive staining, while the PNF group displayed strong, continuous Col I staining along the interface, comparable to the Sham group. Quantitative analysis of the integrated optical density (IOD) (**Figure 5B**) confirmed that Col I expression was significantly higher in the PNF group than in the Model and Traditional groups.

### PNF treatment upregulated VEGF and TGF-β protein expression

**Figure 6:**
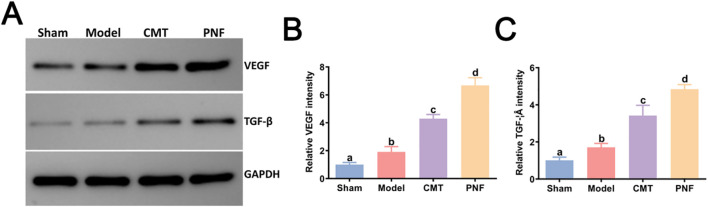
Western blot analysis of growth factor expression. **(A)** Representative Western blot bands of VEGF, TGF-β, and GAPDH. **(B)** Quantitative analysis of VEGF protein expression normalized to GAPDH. **(C)** Quantitative analysis of TGF-β protein expression normalized to GAPDH. Data are presented as mean ± SEM (n = 3 per group). Different letters (a, b, c, d) indicate statistically significant differences at *P* < 0.05 between groups (one-way ANOVA with Tukey’s *post-hoc* test).

Western blot analysis was performed to evaluate the expression of growth factors involved in tendon-bone healing (**Figure 6A**). Quantitative analysis revealed that VEGF expression was significantly reduced in the Model group compared to the Sham group (**Figure 6B**). Both Traditional and PNF treatments significantly increased VEGF levels, with the PNF group showing higher expression than the Traditional group. Similarly, TGF-β expression (**Figure 6C**) was markedly downregulated in the Model group and was restored by both treatments, with the PNF group exhibiting a significantly greater increase compared to the Traditional group.

## Discussion

In this study, we systematically compared the therapeutic effects of PNF versus traditional EA combined with IR on tendon-bone healing in a rat model of rotator cuff tear. Our main findings are as follows: (1) PNF treatment significantly improved the maximum failure load of the tendon-bone interface compared to both the model group and the traditional treatment group; (2) PNF promoted better-organized collagen deposition with a higher type I/type III collagen ratio, indicating enhanced collagen maturation; (3) PNF more effectively suppressed pro-inflammatory cytokine expression (IL-1β, IL-6, TNF-α) at both protein and mRNA levels; (4) PNF upregulated the expression of growth factors VEGF and TGF-β; and (5) PNF improved histological scores for inflammation, tissue architecture, and fibrocartilage regeneration. These results demonstrate that PNF therapy outperforms traditional EA plus IR in promoting rotator cuff healing, providing experimental evidence for its clinical application in chronic rotator cuff injury.

The maximum failure load of the tendon-bone interface is a direct measure of healing quality, as it reflects the integrity and functional capacity of the repaired tissue (Wang et al., 2026). In our study, PNF-treated rats showed a significantly higher failure load than those receiving traditional therapy, and the values approached those of the sham group. This biomechanical advantage is likely attributable to the improved collagen organization and maturation observed in the PNF group, as demonstrated by Masson’s trichrome staining and Sirius red polarization analysis. Previous studies have shown that mechanical loading through passive stretching can enhance collagen fiber alignment and cross-linking, which are critical for tendon-bone healing (Stammers et al., 2020). PNF, with its rhythmic stabilization and resistance components, may provide more physiologically relevant mechanical stimuli than static IR or simple EA, thereby promoting stronger tissue repair (Bano and Aggarwal, 2026).

The histological findings further support the superiority of PNF. Masson’s trichrome staining revealed that PNF-treated rats had densely packed, well-aligned collagen fibers at the tendon-bone interface, whereas the traditional group showed only moderate improvement. Safranin O staining, which visualizes fibrocartilage, demonstrated that PNF promoted more robust regeneration of the transitional zone between tendon and bone. This fibrocartilage layer is essential for stress dissipation and preventing re-tear (Bonnevie and Mauck, 2018). The loss of this zone is a hallmark of chronic rotator cuff tears, and its restoration is considered a key goal of rehabilitation. Our results suggest that PNF, through its multi-directional stretching and muscle activation. In contrast, infrared and EA, while beneficial, may rely more on passive thermal and neural effects without directly addressing the mechanical insufficiency of the healing interface (Locke et al., 2020).

Inflammation plays a dual role in tendon healing: an initial acute inflammatory response is necessary for debris clearance and cell recruitment, but persistent inflammation impairs matrix remodeling and leads to scar formation (Wild et al., 2026). In our study, both treatments significantly reduced the elevated levels of IL-1β, IL-6, and TNF-α induced by rotator cuff tear. However, the PNF group achieved lower cytokine levels than the traditional group, suggesting a more potent anti-inflammatory effect. This may be due to the fact that PNF-induced muscle contractions enhance local blood flow and lymphatic drainage, facilitating the clearance of pro-inflammatory mediators (Aledi et al., 2023). Additionally, mechanical stimulation has been shown to activate anti-inflammatory pathways via integrin signaling and nuclear factor kappa-B (NF-κB) modulation (Lei et al., 2025). This difference may be clinically relevant, as excessive or prolonged inflammation is associated with pain, stiffness, and poor functional outcomes (Quiroz-González et al., 2025).

The growth factor profile further distinguished the two treatments. VEGF is essential for angiogenesis, which delivers oxygen and nutrients to the healing site, while TGF-β is a master regulator of collagen synthesis and tissue remodeling (Jafarbeglou et al., 2026; Song et al., 2026). In our study, both VEGF and TGF-β were significantly upregulated by PNF compared to traditional treatment. This finding aligns with the known mechanobiological response of tendon cells: cyclic stretching and controlled loading upregulate growth factor expression (Nakamichi and Asahara, 2024). PNF, with its repetitive stretch-relaxation cycles and resistance, may provide a more effective mechanical stimulus than the passive heating of infrared or the local neuromodulation of EA (Zhang et al., 2025). The combined increase in VEGF and TGF-β likely contributes to the enhanced vascularity and matrix synthesis observed histologically (Park et al., 2025). Our findings are consistent with recent mechanistic insights showing that mechanical loading regulates enthesis healing through integrin-FAK/MAPK and YAP/TAZ mechanotransduction pathways and promotes collagen I synthesis and matrix remodeling in tendon cells (Squier et al., 2024; Stańczak et al., 2024; Pan et al., 2025).

Several limitations of this study should be acknowledged. Our evaluation was limited to a single time point (4 weeks, late remodeling phase); early and intermediate phases were not assessed, and future multi-time-point studies are needed. Our acute partial tear model in young healthy rats does not fully recapitulate the chronic degenerative rotator cuff tears seen in elderly patients, which limits direct translation to clinical practice. The precise molecular pathways by which PNF modulates inflammation and collagen maturation were not fully elucidated, and functional recovery beyond biomechanics was not assessed.

Despite these limitations, our study provides robust evidence that PNF therapy is superior to traditional EA combined with IR in promoting tendon-bone healing after rotator cuff tear. The advantages of PNF are evident across multiple domains: biomechanical strength, collagen organization and maturation, inflammation resolution, and growth factor expression. These findings support the clinical use of PNF as a first-line non-surgical rehabilitation strategy for chronic rotator cuff injury. Moreover, our results highlight the importance of mechanical loading and neuromuscular re-education in tendon healing, suggesting that passive modalities alone may be insufficient to achieve optimal tissue repair. For patients with partial-thickness tears or those who are not candidates for surgery, PNF offers a safe, non-invasive, and effective approach that addresses both the structural and functional deficits of the injured shoulder (Higgins and Greer, 2025).

## Conclusion

In conclusion, this study demonstrates that PNF therapy significantly improves tendon-bone healing in a rat model of rotator cuff tear, showing superior effects compared to traditional EA combined with IR. PNF treatment enhanced biomechanical strength, promoted well-organized collagen deposition with increased type I/type III collagen ratio, suppressed pro-inflammatory cytokine expression at both protein and mRNA levels, upregulated VEGF and TGF-β, and improved histological outcomes including fibrocartilage regeneration. These findings provide experimental evidence that PNF, as an active neuromuscular rehabilitation approach, offers greater therapeutic benefits than passive physical modalities for chronic rotator cuff injury. Our results highlight the importance of mechanobiological stimulation in tendon-bone healing and suggest that PNF is a promising rehabilitation approach that warrants further clinical investigation.

## Data Availability

The original contributions presented in the study are included in the article/supplementary material. Further inquiries can be directed to the corresponding authors.
